# Attitudes, Beliefs, and Cost-Related Medication Nonadherence Among Adults Aged 65 or Older With Chronic Diseases

**DOI:** 10.5888/pcd15.180190

**Published:** 2018-12-06

**Authors:** Shinduk Lee, Luohua Jiang, Diane Dowdy, Y. Alicia Hong, Marcia G. Ory

**Affiliations:** 1 Texas A & M School of Public Health, College Station, Texas; 2 Texas A & M Center for Population Health and Aging, College Station, Texas; 3 University of California, Irvine, California

## Abstract

**Introduction:**

Cost-related medication nonadherence (CRN) can negatively affect chronic disease prevention and management in an aging population. Limited data are available on the interacting influences among such factors as availability of financial resources, attitudes and beliefs of patients, and CRN. The objective of this study was to examine the causal paths among financial resource availability, patient attitudes and beliefs, and CRN.

**Methods:**

We used a nationally representative sample (n = 4,818) from the 2015 National Health Interview Survey; selected respondents were aged 65 or older, had a diagnosis of hypertension or diabetes or both, and were prescribed medication for at least 1 of these conditions. We performed structural equation modeling to examine whether perceived medication affordability, access to health care, and patient satisfaction influenced the effects of financial resource availability on CRN (skipped doses, took less medicine, or delayed filling a prescription to save money).

**Results:**

Six percent of respondents reported CRN in the previous 12 months. The model showed a good to fair fit, and all paths were significant (*P* < .05) except for age. The effects of financial resource availability on CRN was mediated through perceived medication affordability, access to health care, and patient satisfaction with health care services.

**Conclusion:**

This study suggests that patients’ attitudes and beliefs can mediate the effects of financial resource availability on CRN. We call for senior-friendly public health interventions that can address these modifiable barriers to reduce CRN among older adults with chronic conditions.

## Introduction

Medication nonadherence is a persistent public health issue that can influence management of chronic conditions, especially among older adults (aged ≥65), who are more likely to bear a greater disease burden than their younger counterparts. Although many reasons exist for medication nonadherence, older adults may forgo medications because of cost-related concerns, such as lack of adequate prescription coverage by insurance programs and out-of-pocket costs (1–3). This type of medication nonadherence is called cost-related medication nonadherence (CRN). CRN has been observed among approximately 10% to 40% of noninstitutionalized older adults (4–6). The adverse consequences of medication nonadherence include poorer health, increased risk of mortality, and greater health care costs (7–9).

Beyond financial factors, CRN is also associated with patient-related factors (eg, sociodemographic characteristics, health status, attitudes) (10–13) and the relationship between patients and their health care providers (14). For example, patient satisfaction, an attitude associated with the evaluation of a patient’s experience with health care services (15–17), has a strong positive association with medication adherence (18). Medication affordability is a proximal driver of medication nonadherence in the proximal–distal continuum of adherence drivers (PDCAD) model (19). The PDCAD model is a conceptual framework that maps the determinants of medication adherence along a continuum of impacts, such that the proximal drivers have the strongest effect on medication nonadherence (19). Medication affordability is not necessarily bound by the cost of prescriptions or financial resource availability (20). Additionally, physical access to health care may have some effect, but a small one, on CRN (21,22).

By using the extended PDCAD model (19), we hypothesized that patient satisfaction with health care, physical access to health care, and medication affordability would mediate the relationship between CRN and financial resource availability, age, and mental health status. The objective of this study was to describe the effects of patient attitudes and beliefs on CRN and the mediating effects of patient attitudes and beliefs on the relationship between financial resource availability and CRN.

## Methods

Our data source was the 2015 National Health Interview Survey (NHIS). The NHIS is a cross-sectional household interview survey conducted by the National Center for Health Statistics; it collects health and health-related information via face-to-face interviews among the civilian noninstitutionalized population living in United States (23). We selected participants according to the following self-reported criteria: 1) having a diagnosis of hypertension or diabetes, 2) having prescription medication for those conditions, and 3) being aged 65 or older at the time of the assessment. Proxy-reported data were excluded. We conducted our analysis from April 2017 through April 2018.

### Variables

Data were collected on 3 types of CRN behaviors in the previous 12 months: skipping medication to save money, taking less medicine to save money, or delaying filling a prescription to save money. Each item was scored as yes or no. A participant who answered yes to any of the 3 items was categorized as reporting CRN, and a participant who answered no to all 3 items was categorized as not reporting CRN. Reliability tests of the measures showed high test–retest reliability (κ ≥ 0.6) (24).

We assessed medication unaffordability by using a single item: “During the past twelve months, was there any time when you needed [prescription medicine] but didn’t get it because you couldn’t afford it?” The item was scored as yes or no.

Patient satisfaction with health care services was assessed by using a single item: “In general, how satisfied are you with the healthcare you received in the past twelve months?” Responses ranged from 1 (“very dissatisfied”) to 4 (“very satisfied”). Twenty-six (0.5%) of the 4,818 study-eligible participants did not report on this variable because they had not received any health care in the previous 12 months.

We used data on 5 areas of physical access to health care in the previous 12 months. All 5 items asked about delays in getting health care. Participants were asked about delays because of 1) difficulty in getting through on the telephone, 2) difficulty in getting an appointment in a timely manner, 3) long wait times at the health care setting, 4) the clinic or doctor’s office not being open when the participant could get there, and 5) lack of access to transportation. Each item was scored as yes or no. A participant who answered yes to any of the 5 items was categorized as having poor physical access to care, and a participant who reported no to all 5 items was categorized as not having poor physical access to health care.

We categorized the study sample into 4 groups based on the ratio of family income to the 2014 federal poverty thresholds (<1.00, 1.00–1.99, 2.00–3.99, ≥4.00). A ratio of less than 1 indicates that family income is below the federal definition of poverty (ie, <$11,670 for a household size of 1 in 2014).

The 2015 NHIS used the K6 screening scale for assessing the presence of serious mental illness. The K6 screening scale comprises six 5-point Likert items on how often respondents felt nervous, hopeless, restless or fidgety, depressed, or worthless or needed effort on everything in the past 30 days. For each item, the possible response ranges from 1 (all of the time) to 5 (none of the time); hence, the sum of the 6 items can range from 6 to 30. Using the dichotomous scoring approach described by Kessler et al (25), we classified participants who had a summary score of less than 19 as not having serious mental illness and participants who had a summary score of 19 or higher as having serious mental illness. The Cronbach α for the scale was 0.84.

**Demographic and sociodemographic information.** The 2015 NHIS collected data on age, sex, race, ethnicity, region of residence, education, and self-rated health. We categorized age into less than 75 years and 75 years or older. The dichotomization was guided by distribution of the data, such that about half of the study sample was assigned to each category. Sex was categorized as male and female. Ethnicity was categorized as Hispanic and non-Hispanic. Race was dichotomized as white and nonwhite. Region was classified into Northeast, Midwest, South, and West.

### Analysis

The final study sample consisted of 4,818 adults aged 65 or older with hypertension or diabetes or both, accounting for 11.4% of all 2015 NHIS participants (N = 42,288). We calculated mean and standard deviation or frequency and percentage to describe characteristics of the study participants. We used listwise deletion for handling missing data. Of the 4,818 adults, 1,254 (26%) had missing values for at least one of the variables included in the structural equation model (SEM). We found few missing values (<5%) for most variables, but 85% (1,063 of 1,254) of participants who had missing values did not report all the income-related information to estimate the financial resource availability. Because of this missing information, we tested the final hypothesized model by using alternative measures of financial resources (eg, ever concerned about not having enough food in the past 12 months) that had lower rates of missing data. Although we did not tabulate these data for this study, we found no meaningful difference between the analysis using the income variables and the analysis using the alternative variables. By using χ^2 ^tests, we found a positive association with having 1 or more missing values for the SEM variables for being 75 or older, female, or nonwhite; living in the Midwest; not having poor physical access to health care; or having a serious mental illness. Similarly, being 75 or older, female, or nonwhite or living in the Midwest were positively associated with missing data on financial resource availability. Using quantile regression for interval variables and χ^2 ^tests for categorical variables, we compared study participants who reported CRN with study participants who did not report CRN.

**Figure 1:**
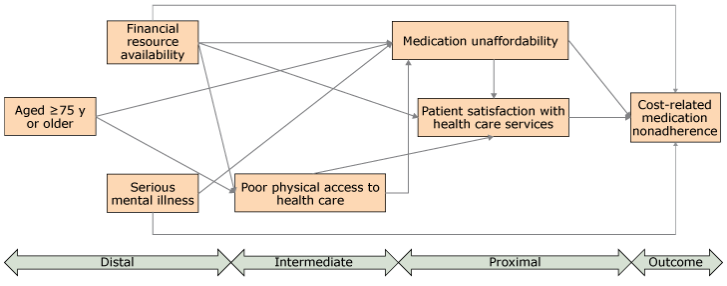
Hypothesized model depicting factors influencing cost-related medication nonadherence (CRN) among adults aged 65 years or older, National Health Interview Survey, 2015. The final hypothesized model included 3 exogenous variables (aged 75 or older, serious mental illness, and financial resource availability) and 4 endogenous variables (CRN, patient satisfaction with health care services, poor physical access to health care, and medication unaffordability). Arrow indicates “effects on”; for example, effects of poor physical access to health care on medication unaffordability.

After conducting the preliminary analyses, we used the SEM to examine patient attitudes and beliefs as potential mediators of the relationship between financial resource availability and CRN. We followed conventional SEM steps: model identification, parameter estimation, fit evaluation, and model re-identification. We used the robust maximum likelihood estimation method to account for nonnormal categorical endogenous variables. SEM can also evaluate fit of the mediated model relative to unmediated and saturated models. We used the χ^2 ^test, the confirmatory factor index, the root mean square error of approximation (RMSEA), and the standardized root mean square residual (SRMR) for the fit evaluation. We considered the following to indicate a good fit: a nonsignificant (*P* ≥ .05) χ^2 ^test result, a confirmatory factor index greater than 0.95, an RMSEA less than 0.05, and a SRMR less than 0.05. We considered an RMSEA between 0.05 and 0.08 to indicate a fair fit. Furthermore, we used modification indices to identify potential areas of the model that had a poor fit. The direct path between physical access to care and CRN was dropped to obtain the better fit. The final model for testing included 3 exogenous variables (aged 75 or older, serious mental illness, and financial resource availability) and 4 endogenous variables (CRN, patient satisfaction with health care, poor physical access to care, and medication unaffordability) (Figure 1).

We performed all statistical analyses in SAS version 9.4 (SAS Institute Inc). As instructed in the NHIS data guide, we conducted the subset analyses by using the complete data file to ensure correct estimation of variance and use of appropriate sampling weights. We confirmed with the Texas A & M University institutional review board that the study did not require approval because it used a public use data set.

## Results

The median age of the study sample was 73, and most participants were female (54.7%), non-Hispanic (91.7%), and white (83.0%) (Table 1). Of 4,818 participants, 269 (5.9%) (weighted percentage, 5.7%) reported CRN in the previous 12 months. Compared with those who did not report CRN, those who reported CRN were younger (71 y vs 73 y), were more likely to be female (61.3% vs 54.0%) and have had a diagnosis of both hypertension and diabetes (36.5% vs 26.7%), and were less likely to be white (78.6% vs 83.7%), non-Hispanic (89.4% vs 92.0%), and married or living with a partner (47.8% vs 56.7%). Responses to the question on satisfaction with health care services were highly skewed, such that 72.7% reported being very satisfied with health care services.

**Table 1:** Characteristics of a Sample of Participants (n = 4,818) in Study on Cost-Related Medication Nonadherence^a^^,^ b ^,^ c Among Adults Aged 65 Years or Older

Characteristic	Overall		Cost-Related Medication Nonadherence,^b^ Weighted^d^ %		*P* Value^e^
	Unweighted No. (%)	Weighted^d^ %	Yes	No	
**Age, y^c^**	73	73	71	73	.006
**Sex**					
Female	2,828 (58.7)	54.7	61.3	54.0	<.001
Male	1,990 (41.3)	45.3	38.7	46.0	
**Ethnicity**					
Non-Hispanic	4,343 (90.1)	91.7	89.4	92.0	<.001
Hispanic	475 (9.9)	8.3	10.6	8.0	
**Race**					
White	3,814 (79.2)	83.0	78.6	83.7	<.001
Black or African American	713 (14.8)	11.1	14.7	10.4	
Asian	177 (3.7)	4.0	3.0	4.2	
Other races	38 (0.8)	0.6	1.6	0.5	
Multiple races	76 (1.6)	1.3	2.1	1.3	
**Marital status**					
Married or living with a partner	1,972 (40.9)	55.9	47.8	56.7	<.001
Not married or living with a partner	2,846 (59.1)	44.1	52.2	43.3	
**Diagnosis**					
Hypertension only	3,290 (68.3)	68.1	59.2	68.5	<.001
Diabetes only	214 (4.4)	5.0	4.4	4.8	
Both hypertension and diabetes	1,312 (27.2)	27.0	36.5	26.7	
**Reported cost-related medication nonadherence^b^**	269 (5.9)	5.7	—f	—f	—f

a Data source: 2015 National Health Interview Survey (23).

b Data were collected on 3 types of cost-related medication nonadherence behaviors in the previous 12 months: skipping medication to save money, taking less medication to save money, or delaying filling a prescription to save money. Each item was scored as yes or no. A participant who answered yes to any of the 3 items was categorized as reporting CRN, and a participant who answered no to all 3 items was categorized as not reporting CRN.

c Units in column heads apply to cells in all rows, except for row indicating age, which is reported in years.

d Weighted by sampling weights.

e Comparison between participants who reported CRN and participants who did not report CRN. Age was compared by using quantile regression, and categorical variables were compared by using χ^2^ tests.

f Not applicable.

The tested model had a good to fair fit based on the 3 fit indices: a confirmatory factor index of 0.97, an RMSEA of 0.06, and an SRMR of 0.03. The χ^2^ test of fit was significant (χ^2^_5_ = 66.7; *P* < .001), indicating a poor fit. However, this test can be sensitive to trivial deviations from the perfect fit with a sufficiently large sample size. Despite the adequate fit of the model, the model explained only 34.3% of the variance in CRN.

**Figure 2:**
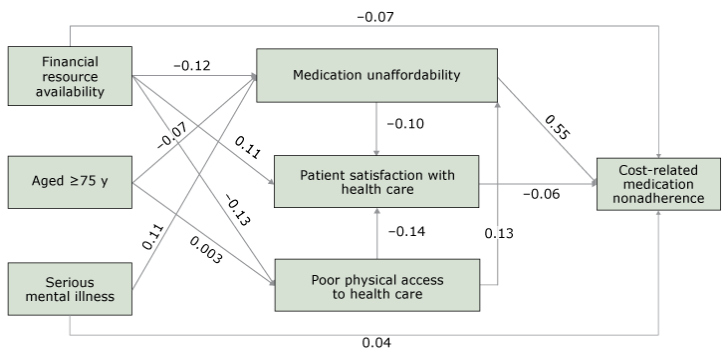
Path diagram of the final structural equation modeling among adults aged 65 years or older, National Health Interview Survey, 2015. The path diagram describes the magnitudes and significance of the hypothesized relationship between age, serious mental illness, financial resource availability, patient’s attitudes and beliefs, and cost-related medication nonadherence (CRN). Arrow indicates “effects on”; for example, effects of poor physical access to health care on medication unaffordability.

A greater likelihood of CRN was associated with greater medication unaffordability (β = 0.55; standard error [SE], 0.01; *P* < .001), lower levels of patient satisfaction with health care (β = −0.06; SE, 0.01; *P* < .001), less financial resource availability (β = −0.07; SE, 0.01; *P* < .001), and serious mental illness (β = 0.04; SE, 0.01; *P* = .001). Medication unaffordability was associated with poor physical access to health care (β = 0.13; SE, 0.02; *P* < .001), lower levels of financial resource availability (β = −0.12; SE, 0.02; *P* < .001), serious mental illness (β = 0.11; SE, 0.02; *P* < .001), and being younger than 75 (β = −0.07; SE, 0.02; *P* < .001). Lower levels of patient satisfaction were associated with greater medication unaffordability (β = −0.10; SE, 0.02; *P* < .001), poorer physical access to health care (β = −0.14; SE, 0.02; *P* < .001), and less financial resource availability (β = 0.11; SE, 0.02; *P* < .001). Poor physical access to health care was negatively associated with financial resource availability (β = −0.13; SE, 0.02; *P* < .001). Effect of age on physical access to health care was not significant (β = 0.003; SE, 0.02; *P* = .84) (Figure 2).

Medication unaffordability had the strongest total effects on CRN, followed by financial resource availability, serious mental illness, poor physical access to health care, and patient satisfaction with health care services (Table 2). Age had the least total effect on CRN. Approximately half of the effects of financial resource availability on CRN were mediated through medication unaffordability, poor physical access to health care, and patient satisfaction with health care (direct effects = −0.072; indirect effects = −0.084).

**Table 2:** Standardized Total, Direct, and Indirect Effects in the Hypothesized Structural Equation Model of Cost-Related Medication Nonadherence (CRN) Among Adults Aged 65 Years or Older^a^^,^ b

Predictor	Mediated Through . . .	Causal Effect^c^		
		Total	Direct	Indirect
Medication unaffordability^d^	No mediator	—	0.550	—
	Patient satisfaction with health care	—	—	0.006
	Total	0.556	0.550	0.006
Financial resource availability^e^	No mediator	—	−0.072	—
	Medication unaffordability	—	—	−0.067
	Patient satisfaction with health care	—	—	−0.007
	Medication unaffordability → patient satisfaction with health care	—	—	−0.001
	Poor physical access to health care → medication unaffordability	—	—	−0.009
	Poor physical access to health care → medication unaffordability → patient satisfaction with health care services	—	—	<0^f^
	Poor physical access to health care → patient satisfaction with health care	—	—	−0.001
	Total	−0.156	−0.072	−0.084
Serious mental illness^g^	No mediator	—	0.044	—
	Medication unaffordability	—	—	0.059
	Medication unaffordability → patient satisfaction with health care services	—	—	0.001
	Total	0.103	0.044	0.059
Poor physical access to health care^h^	Patient satisfaction with health care	—	—	0.009
	Medication unaffordability	—	—	0.069
	Medication unaffordability → patient satisfaction with health care services	—	—	0.001
	Total	0.079	—	0.079
Patient satisfaction with health care services^i^	No mediator	—	−0.063	—
	Total	−0.063	−0.063	—
Aged ≥75 y	Medication unaffordability	—	—	−0.038
	Medication unaffordability → patient satisfaction with health care services	—	—	<0^f^
	Total	−0.038	—	−0.038

Abbreviation: →, effects on.

a Data source: 2015 National Health Interview Survey (23).

b Data were collected on 3 types of CRN behaviors in the previous 12 months: skipping medication to save money, taking less medication to save money, or delaying filling a prescription to save money. Each item was scored as yes or no. A participant who answered yes to any of the 3 items was categorized as reporting CRN, and a participant who answered no to all 3 items was categorized as not reporting CRN.

c Direct effect refers to the direct relationship between the 2 variables (ie, predictor variable and outcome variable). Indirect effects refer to the effect of a predictor variable on an outcome variable via one or more mediator variables. Total effect of a predictor variable on an outcome variable is the sum of direct and indirect effects. For example, approximately half of the total effects of financial resource availability on CRN were the indirect effects through medication unaffordability, poor physical access to health care, and patient satisfaction with health care services.

d Assessed by using single yes–no item: “During the past twelve months, was there any time when you needed [prescription medicine] but didn’t get it because you couldn’t afford it?”

e We categorized the study sample into 4 groups based on the ratio of family income to the 2014 federal poverty threshold (<1.00, 1.00–1.99, 2.00–3.99, ≥4.00).

f Less than 0, but greater than −0.001.

g Assessed by Kessler’s K6 screening scale and dichotomous scoring approach (25).

h Five yes–no items asked about delays in getting health care in previous 12 months: delays because of 1) difficulty in getting through on the telephone, 2) difficulty in getting an appointment in a timely manner, 3) long wait times at the health care setting, 4) the clinic or doctor’s office not being open when the participant could get there, and 5) lack of access to transportation. A participant who answered yes to any of the 5 items was categorized as having poor physical access to care, and a participant who reported no to all 5 items was categorized as not having poor physical access to health care.

i Assessed by using a single item: “In general, how satisfied are you with the healthcare you received in the past twelve months?” Responses ranged from 1 (“very dissatisfied”) to 4 (“very satisfied”).

## Discussion

Approximately 6% of our sample of adults aged 65 or older with diabetes or hypertension or both reported CRN. This prevalence of CRN is similar to the 8% reported in the Health, Aging and Body Composition Study (26), but it is lower than the prevalence reported in other studies (4–6). CRN could be influenced by multiple factors (eg, disease type, disease duration), which might explain the broad range of CRN rates observed among older adults. Our study demonstrated that the effects of financial resource availability on CRN were mediated through patient’s attitudes and beliefs on such factors as medication unaffordability, physical access to health care, and patient satisfaction with health care services. Our study expands understanding of the mechanisms driving CRN among older adults and suggests potential intervening points for enhancing CRN in this population.

Our findings are in line with the findings of studies showing that the relationship between financial pressure and CRN is complex and that financial pressure alone cannot explain CRN. For example, one study (27) observed CRN in both low-income and high-income populations and found that having low levels of concern about medication use (eg, side effects) was associated with lower levels of CRN in both populations. The difference between that study and our study is that the former focused on the independent effects of patients’ attitudes on CRN by the degree of financial pressure, whereas our study focused on the causal paths that link these multiple factors together.

As hypothesized under our conceptual framework, medication unaffordability was the strongest predictor of CRN, and this finding is consistent with previous research (19,28). One study of a national sample of adults with at least 1 chronic condition found that medication affordability was the most common reason for medication nonadherence (28). More than half of study respondents who engaged in medication nonadherence reported that medication affordability was their reason for nonadherence (28). Another study found that adults who had the lowest levels of perceived medication affordability had a significantly greater likelihood of medication nonadherence than those with the highest levels of perceived medication affordability (19). Our study confirms these previous findings and strengthens the evidence on reasons for CRN among older adults.

Our study showed that patient satisfaction with health care services was adversely associated with CRN. Similarly, another study indicated a weak positive correlation between patient satisfaction and medication adherence among patients with HIV/AIDS (*r* = 0.178, *P* < .05) (29). However, that study did not examine the independent effects of patient satisfaction on medication nonadherence, and the study population was different from the population in our study.

As hypothesized in the PDCAD model, age had weaker effects on CRN than did other variables in the model. This observation is not surprising given the conflicting findings of previous studies (30,31). For example, in 2 studies of medication adherence to adjuvant hormonal therapy among breast cancer patients, 1 study indicated that older age (>70 y) was a potential barrier to medication adherence (30), whereas the other study indicated that younger age (<70 y) was a potential barrier (31).

Our study has several limitations. First, the cross-sectional data precluded examination of cause and effect. However, previous studies support the hypothesized causal paths among financial resource availability, patient attitudes and beliefs, and CRN. Second, our study relied on self-reported data, which are subject to social desirability bias and recall bias, and these data could not be verified. However, self-reported data on medication nonadherence can provide contextual information and enable researchers to identify types of medication nonadherence (eg, intentional vs unintentional, cost-related vs not cost-related). Third, this survey had only a single item on patient satisfaction with health care, and responses to the items were highly skewed. Fourth, our model did not include some potentially important drivers, such as perceived need for medication and perceived concerns about medication use. The model explained only about one-third of the variance of CRN, and the inclusion of other important determinants could improve the model’s explanatory power. Finally, we used listwise deletion for handling missing data, and we omitted records for more than 25% of the participants by using this approach. Study participants who did not report any problems with physical access to health care were more likely to be omitted from the analysis, and, therefore, the likelihood of having access to health care services was potentially biased downward. In addition, using listwise deletion tends to result in large standard errors and wide confidence intervals. To overcome this limitation, we tested the final model by using alternative measures of financial resources (eg, concerns about running out of food) that had low rates of missingness, and we found no meaningful change in the results (ie, no changes in significance and minimal changes in regression coefficients).

Despite these limitations, our study has some strengths. Our study is one of the few studies that attempted to explore the causal paths between financial resource availability and CRN and to understand the potential effects of patient attitudes and beliefs on the relationship. Another strength is that we used a nationally representative data set.

Our study aimed to explain the relationship among financial resource availability, patients’ attitudes and beliefs (eg, perceived medication affordability, physical access to health care, and patient satisfaction with health care services), and CRN, a relationship that had not been examined previously. Our findings suggest that perceived medication affordability is the key driver of CRN and that the effects of financial resource availability on CRN is mediated through medication affordability, physical access to health care, and patient satisfaction with health care services. Although several possible pharmaceutical policy reforms (eg, the involvement by government in determining the prices of prescription drugs) should be considered in addressing the financial-related factors that drive medication nonadherence, many uncertainties exist at this stage of health policy reform. Meanwhile, our study suggests that rates of CRN among older adults can be reduced by decreasing financial pressure as well as by modifying attitudes and beliefs among this population. It also points to the need for public health interventions that address these modifiable barriers for older adults. Future research should test more comprehensive models by including other important attitudinal variables, such as the perceived need for medications and concern about their use. Future models should also include factors related to health care providers, prescriptions, and the health care system.
